# Utility of dominant epitopes derived from cell-wall protein LppZ for immunodiagnostic of pulmonary tuberculosis

**DOI:** 10.1186/s12865-018-0243-2

**Published:** 2018-03-01

**Authors:** Jinjing Tan, Xiaoguang Wu, Suting Chen, Meng Gu, Hairong Huang, Wentao Yue

**Affiliations:** 1 Department of Cellular and Molecular Biology, Beijing Chest Hospital, Capital Medical University/Beijing Tuberculosis and Thoracic Tumor Research Institute, Beijing, 101149 China; 2 Department of Tuberculosis, Beijing Chest Hospital, Capital Medical University, Beijing, 101149 China; 3 National Clinical Laboratory on Tuberculosis, Beijing Key laboratory on Drug-resistant Tuberculosis Research, Beijing Chest Hospital, Capital Medical University/Beijing Tuberculosis and Thoracic Tumor Institute, Beijing, 101149 China; 4 Central Laboratory, Beijing Obstetrics and Gynecology Hospital, Capital Medical University, Chaoyang, Beijing, 100026 China

**Keywords:** Tuberculosis peptide arrays, LppZ, Serologic test

## Abstract

**Background:**

Serological antibodies tests for tuberculosis (TB) are widely used in developing countries. They appear to have some advantages- faster, simple and could be used for extrapulmonary TB. However, most of current commercial TB serological tests are failed to provide sufficient sensitivity and specificity. Improved serological biomarkers were essential. In this study, we present an approach using peptide array to discover new immunodiagnostic biomarkers based on immunodominant epitopes of TB antigens.

**Results:**

The Probable conserved lipoprotein LppZ, which is difficult to express and purify in vivo was selected as the model antigen. We use two-step screening for dominant epitope selection. Based on peptide array data from 170 TB patients and 41 control samples, two dominant epitopes were identified to have diagnostic value for TB patients. Truncation assay was used to identify the core reactive sequence. Peptide- based ELISA was used to evaluate the diagnostic ability of pep-LppZ-1 and pep-LppZ-13. Pep-LppZ-1 has a sensitivity of 49.2% and a specificity of 83.3% in TB diagnose. Pep-LppZ-13 has a sensitivity of 43.3% and a specificity of 88.5% in TB diagnose.

**Conclusions:**

Our result demonstrated that peptide array screening would be an advantage strategy of screening TB diagnostic peptides.

**Electronic supplementary material:**

The online version of this article (10.1186/s12865-018-0243-2) contains supplementary material, which is available to authorized users.

## Background

Tuberculosis (TB) ranks as the second major cause of death among infectious diseases around the world. In 2014, 9.6 million people around the world became sick with TB disease. There were 1.5 million TB-related deaths worldwide. Only 66% of the TB-cases worldwide are correctly diagnosed [1]. China is one of the 22 high TB burden countries in the world, second only to India [2]. About 550 million people was infected with *Mycobacterium tuberculosis* (*Mtb*). Early diagnosis of TB is especially important for improving early treatment. The gold standard in TB diagnosis remains to be the bacteria culture from sputum or body fluid specimens, which is time-consuming and low efficiency.

Serological tests that rely on the detection of TB-specific antigens or antibodies against TB-specific antigens possess several advantages: simple, inexpensive and feasible for the diagnosis of TB compared with traditional bacteria culturing. Moreover, it could be used to diagnose extrapulmonary TB. Many TB-specific antigens have been evaluated and applied to develop serological assays [3–5]. However, based on meta-analysis of commercial TB serological test from 1990 to 2010, current TB serological assays were failed to provide sufficient sensitivity and specificity [6]. In China, the diagnosis accuracy of these serological tests was major limited by reduced specificity. The low specificity can be attributed to cross-reactivity with other mycobacteria involving environment *Mtb* exposure. World Health Organization (WHO) warned against the use of current inaccurate serological test for TB clinic diagnose [7]. The WHO expert group strongly encouraged further study on identifying improved serological tests.

From the previous proteomic studies, many *Mtb* proteins can be detected by serum antibodies [8]. However, due to complicated post-translational modification, most *Mtb* proteins were difficult to express and purify [9]. For the matter of fact, epitopes of *Mtb* proteins are essential for the antibody reaction to reach the diagnostic accuracy. Protein array research revealed that several protein fragments from bacteria culture filtrate or protein lysate were immunoreactive with serum antibodies [10, 11]. Therefore, instead of expressing recombinant antigens, using epitope peptides is an alternative approach to develop novel biomarkers of serodiagnosis [12]. Recent studies demonstrated that peptide epitopes may even improve the detection efficiency in diagnostic assays for TB [13]. High-throughput proteomic study could help to screen diagnostic antigens in form of peptides or protein fragments that contain immunodominant epitopes. In this context, combinatorial phage display has emerged as a direct method for discovering novel antigens [14–16]. Since high quality peptides are easy to access through chemical synthesis, peptide arrays become an advanced technology. Without translating DNA to protein, designed libraries of peptides display on solid surface usually a cellulose membrane or a glass chip [17]. Systematically screening immunoreactive peptides for serodiagnosis of TB provided large data of specific TB epitopes [18]. These data also indicated that the segregation between TB and healthy individuals does not cluster into specific proteins, but into specific peptide epitope ‘hotspots’ at different location of the same protein [19]. Therefore, there needs large clinical specimens to validate the candidate peptides, which were screened out by peptide arrays.

Antigen array performed on TB protein fractionations revealed several TB lipoprotein fractionations including LppZ could be recognized by serum antibodies from TB patients [11]. LppZ(Rv3006) is a cell-wall lipoprotein that contains a post-translational modification involved in glycosylation. Expressing recombinant LppZ antigen is time-consuming and challenged by the need for correct folding and modification. An alternative approach of constructing a high-content peptide array, which displays the LppZ antigen in the form of linear peptide could be used to obtain anti-peptide antibodies in sera. These peptides contained dominant epitope sequence that could recognize by serum IgG.

Therefore, we utilize cell-wall protein LppZ as an example, through two-step screening on peptide array to characterize the immunogenicity of LppZ and identify novel antigens as potential candidates for serological diagnosis of tuberculosis. Here, the entire LppZ protein sequence were dissembled and synthesized on cellulose membrane for IgG reactive epitope mapping. Reactive epitopes ware evaluated by ELISA assay and truncation assay.

## Results

### LppZ epitope mapping using peptide array

To identify the serum IgG-binding epitope, a set of 121 different 15-mer peptides generated from LppZ epitope mapping library was synthesized on cellulose membranes for the first round screening. Each peptide was shift 3 amino acids from its neighboring peptide. We used serum pools, which contained equivalence mixture of 10 serum samples from TB patients for immunoblotting (Additional file 1: Figure S1). A total 16 immunoreactive spots were obtained after first round screening.

### Second round of screening for dominant epitopes

**Fig. 1:**
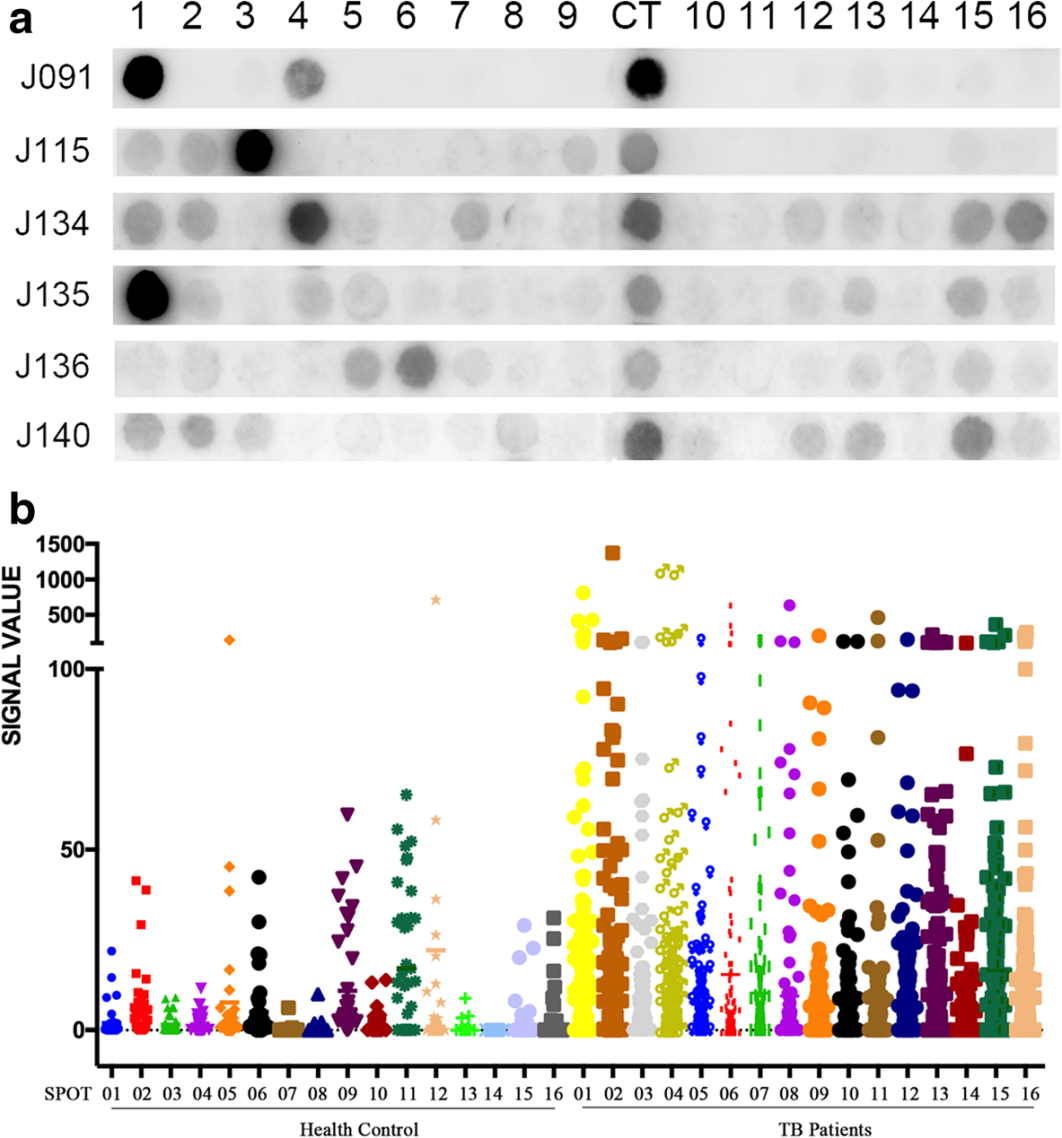
Screening for LppZ dominated epitopes. **a** Second round peptide array screen of 16 candidate epitope peptides selected from first round screening. J091 to J140 stands for serum sample name; CT represents positive control. **b** Scattergram of peptide arrays signals. Data were normalized by CT signal on each array. Full-length blots are presented in Additional file 1: Figure S1

Then those 16 selected IgG-bond epitopes were integrated on a small array for the second round of screening of dominated epitope. A large-scale of individual serum samples of TB patients and health control were used in immunoblotting (Fig. 1a). The clinical character of TB patients was sorted in Additional file 1: Table S1. In order to normalize between arrays, we spotted FLAG-tag peptide as a positive reference probe on the array membrane. Data of each array was generated by image analysis software including spot gray value and background gray value. The signal value was created after normalization transformation including background correction and baseline conversion. The signal values of 16 spots immunoblotting with 170 TB patients and 41 health controls were summarized in Fig. 1b. The sequence and statistic characteristics of each spot were summarized in Additional file 1 Table S2 & S3.

The criteria for selecting peptides with diagnostic potential included a) high ability to react with TB serum sample; b) low ability to react with health control serum sample; c) statistically significant difference between reactivity with TB serum and health controls. AUC (area under the curve) of ROC (Receiver operator characteristic curve) curve of each spot was also under consideration. Based on these criteria, pep-LppZ-1 and pep-LppZ-13 were selected as dominated epitopes for further studies. The positive reactivity rate of pep-LppZ-1 and pep-LppZ-13 among the TB patients were 20.6% (35/170) and 15.3% (26/170) respectively. Both pep-LppZ-1 and pep-LppZ-13 were negative reactivity among health controls. Both AUC of pep-LppZ-1 and pep-LppZ-13 were above 0.7, which indicated a reliable discriminatory ability between TB and health group on chip.

### Core sequence identification

Truncation peptide libraries of selected peptides were constructed to identify the shortest amino acid essential for antigen-antibody interactions. To narrow down the core activity sequence, the library is constructed by systemically removing the flanking residues of the original peptide. While the essential amino acid was truncated, the peptide was failed to interact with the antibody.

**Fig. 2:**
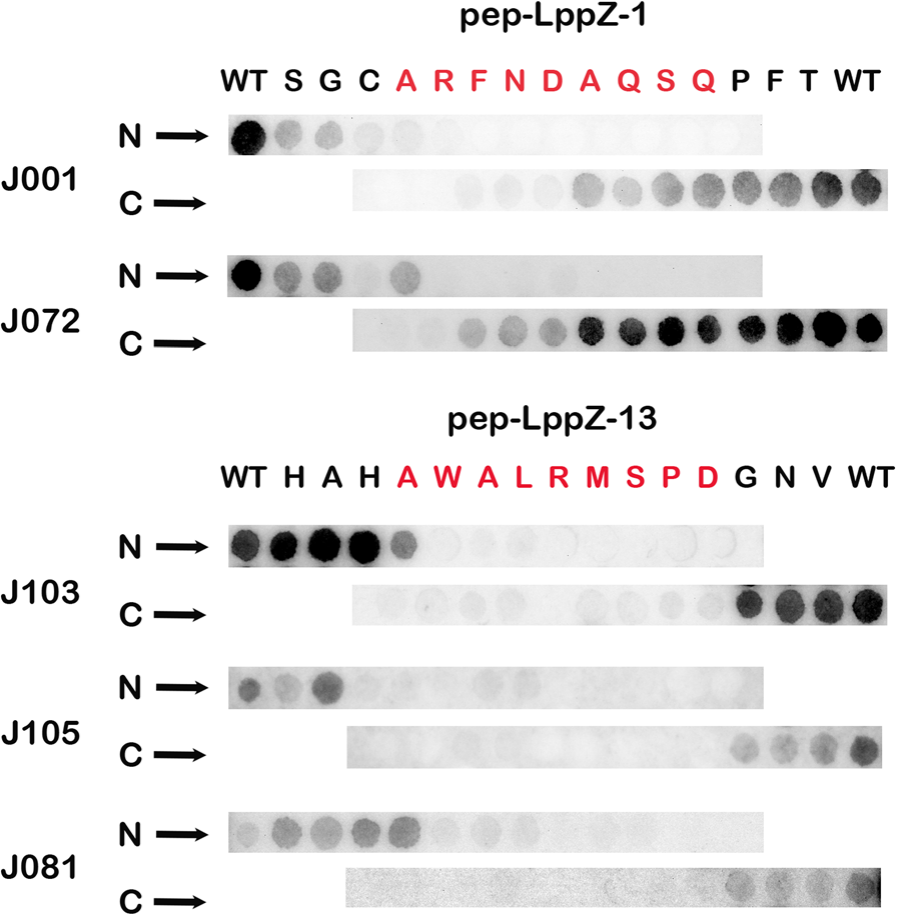
Identify the core sequence of candidate peptides during serum antibody reaction. A: Truncation assay on pep-LppZ-1 and pep-Lppz-13 peptides. N➡ indicates the truncation starts at the N terminal; C ➡ indicates the truncation starts at the C terminal. WT stands for wide type. Each letter means the amino acid was removed. Full-length blots are presented in Additional file 1: Figure S2

For two of the dominant peptides identified from the screening array, the key interaction residues were located at N terminus of pep-LppZ-1 and middle of pep-LppZ-13. When cutting the first amino acid from the N terminal of pep-LppZ-1, the peptide failed to react with the serum antibodies. The reactivity ability was not affected until the eighth amino acid of pep-LppZ-1 was removed from C terminal. The pep-LppZ-13 failed to interact with serum antibodies when either removes the fifth amino acid from N terminal or the fourth amino acid from C terminal (Fig. 2). As the result showed the core sequence of pep-LppZ-1 and pep-LppZ-13 were A-R-F-N-D-A-Q-S-Q and A-W-A-L-R-M-S-P-D, respectively.

### Validation and evaluation by ELISA

**Fig. 3:**
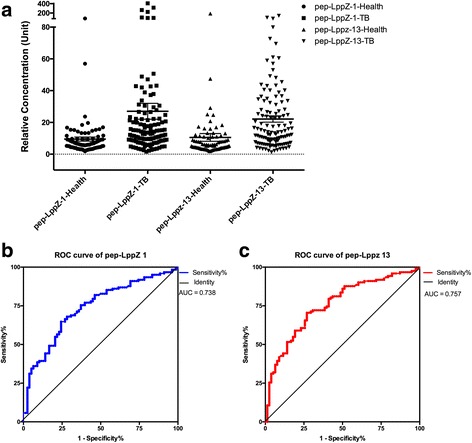
Validate and evaluate the reactivity of dominated epitopes by ELISA. **a** Histogram of relative concentration of antibodies toward dominated epitopes. **b** & **c**: ROC (receiver operating characteristic) curve of pep-LppZ-1 and pep-LppZ-13 on detecting TB infection based on ELISA data

The reactivity of dominated epitopes was validated by ELISA assay. KLH-peptide conjugates on microtiter plates were tested with 170 TB serum samples and 78 health controls. An equivalence mixed serum pool was served as standards using gradient dilution methods. The IgG levels of serum sample were calculated according to the standard curve as relative concentration to serum pool, which was 10. The statistic characteristics of relative concentration of peptide detected IgG are illustrated in Table 1. In accordance with the peptide array result, the IgG reactivity against peptide pep-LppZ-1 and pep-LppZ-13 was significantly higher in the TB patients group when compared to the health control group. The difference between TB patients and health controls was statistically significant (*p* < 0.001) for both of the peptides (Fig. 3a).

**Table 1:** Statistic characteristics of pep-LppZ-1 and pep-LppZ-13 based ELISA

Peptide name	TB patients (*n* = 122)		Health Controls (*n* = 78)		*P* value^1^	AUC^2^
	Mean ± SD	Sensitivity	Mean ± SD	Specificity		
LppZ-1	27.03 ± 55.04	49.2%	9.49 ± 11.28	83.3%	< 0.001	0.738
LppZ-13	22.14 ± 22.11	43.4%	10.55 ± 21.77	88.5%	< 0.001	0.757
Combine		39.3%		91.0%		

^1^*P* value: comparison of difference between TB group and health control group using Mann-Whiney U test

^2^AUC: area under the curve according to receiver operating characteristic (ROC) indicating the discriminatory ability for TB detection from test

### The diagnostic value of dominant epitope

ROC curves for these peptides for TB patients with the control group as reference were plotted (Fig. 3b & c). The AUC for pep-LppZ-1 and prp-LppZ-13 were 0.738 and 0.757 respectively, indicating their discriminatory ability for TB infection detection from test group. When cut-off value for pep-LppZ-1 is > 13.5, the overall sensitivity and specificity of the ELISA test are 49.2% and 83.3%. When cut-off value for pep-LppZ-13 is > 16.3, the overall sensitivity and specificity of the ELISA test are 43.4% and 88.5% (Table 1). Combinative use of two peptides could improve specificity to 90.0% with sensitivity of 39.9%.

Since BCG vaccination is considered as the main interference factor in TB serological diagnose, we compared the diagnose rate of pep-LppZ peptides in BCG vaccination positive and negative groups. The diagnostic sensitive showed not much difference between BCG vaccination positive and negative groups (Table 2). The result suggested that the peptide diagnose value would not be disturbed by individual BCG vaccination condition.

**Table 2:** Relationship between BCG vaccination and peptide diagnose

		pep-LppZ-1		pep-LppZ-13	
Subgroups	N	Positive N (Cutoff > 13.5)	Positive rate	Positive N (Cutoff > 16.3)	Positive rate
Health control (IGRA-)^1^					
BCG+	11	3	27.3%	1	9.1%
BCG-	20	4	20.0%	2	10.0%
TB patient					
BCG+	93	45	48.4%	39	37.5%
BCG-	8	4	50.0%	3	41.9%

^1^IGRA: IFN-γ release assay (T-SPOT.TB); ^2^BCG: Bacillus Calmette- Guerin vaccine

We then analyze the diagnostic consistency of peptide based ELISA and traditional methods including AFB stain (Acid Fast Bacilli stain) and IGRA test (T-SPOT) (Interferon Gamma Release Assay) (Table 3). In TB diagnose, the judgment of peptide ELISA has 60.8% overlap with IGRA test, 44.6–46.4% overlap with sputum smear and 50.5–51.5% overlap with bacteria culture. When comparing the sensitive of peptide ELISA in TB diagnose between smear/ culture/ IGRA positive group and smear/ culture /IGRA negative group, it hardly showed bias, which revealed that the mechanism of peptide diagnoses differ from that of traditional methods (Table 4). Hence, using peptide ELISA as a complement would greatly improve the detection rate of TB (Table 5).

**Table 3:** Diagnose consistency of peptides and traditional methods

	N	pep-LppZ-1 (cutoff > 13.5)	pep-LppZ-13 (cutoff > 16.3)
Pep-LppZ-1 vs. pep-LppZ-13	122	87.5%	–
IGRA test vs.	171	60.8%	60.8%
Sputum smear vs.	56	46.4%	44.6%
Bacteria Culture vs.	99	51.5%	50.5%

**Table 4:** Comparison of using peptides based ELISA and traditional methods in TB diagnose

		pep-LppZ-1		pep-LppZ-13	
TB Subgroups	N	Positive N (Cutoff > 13.5)	Sensitive	Positive N (Cutoff > 16.3)	Sensitive
Smear^1^ +	48	22	45.8%	20	41.7%
Smear -	8	4	50.0%	3	37.5%
Culture^2^ +	88	46	52.3%	43	48.9%
Culture -	11	6	54.5%	4	36.4%
IGRA^3^ +	80	38	47.5%	32	40.0%
IGRA -	13	8	61.5%	6	46.2%

^1^Smear: sputum smear detection for TB bacteria

^2^Culture: bacteria culture

^3^IGRA: IFN-γ release assay (T-SPOT.TB)

**Table 5:** Improvement of detection rate by using pep-LppZ-1 and pep-LppZ-13

Diagnose method	N	Positive N	Detection rate
Sputum smear	122	48	39.3%
Smear + pep-LppZ-1^1^		86	70.5%
Smear + pep-LppZ-13^2^		81	66.4%
Smear + pep-LppZ-1/13		89	73.0%
Bacteria culture		88	72.1%
Culture + pep-LppZ-1		102	83.6%
Culture + pep-LppZ-13		98	80.3%
Culture + pep-LppZ-1/13		102	83.6%
IGRA test	93(29 missing)	80	86.0%
IGRA test + pep-LppZ-1		88	94.6%
IGRA test + pep-LppZ-13		86	92.5%
IGRA test + pep-LppZ-1/13		89	95.7%

^1^the threshold of pep-LppZ-1 detection is > 13.5

^2^the threshold of pep-LppZ-13 detection is > 16.3

## Discussion

This study was focused on the use of peptide array screening to discover novel serological biomarkers for TB diagnose. The cell-wall protein LppZ was utilized as an example for proving the possibility of synthetic peptides to improve the early diagnosis of patients infected with TB. Even though there will be post-translation modifications of LppZ protein such as amidation, glycosylation, and phosphorylation or other modifications, previously studies have shown that all the isoforms of LppZ protein fragments were serologically reactive, suggesting that the epitopes recognized by serum antibodies are on the peptide backbone of the core protein and not on probable modification sites [10]. Therefore, overlap mapping was performed on LppZ protein basic sequence regardless of post-translation modification.

In the process of screening, we designed two-step approach for higher probability of success. At the first round of screening, we performed epitope mapping based on the entire LppZ sequence to obtain serum IgG-bound epitopes. The second round of screening was performed by large group of individual serum samples. Based on the peptide array’s result, the diagnostic capability of each candidate epitope was given out at certain diagnostic value (Additional file 1: Table S3). After two rounds of screening, two dominant epitopes, pep-LppZ-1 and pep-LppZ-13, were selected according to our selection criteria. Truncation assay revealed that the core reaction sequence was narrow down to 8 mer amino acids for each peptide. However, when the last unnecessary amino acid was deleted, the binding affinity has been reduced (Fig. 2). Therefore, protective amino acids were needed for immunoreaction.

Then we used traditional serology test method, ELISA to validate the diagnostic capability of these peptides in another group of serum samples, which matches the sample size of peptide array screening. The ELISA result agreed with the peptide array data in terms of diagnostic ability. Compare to other research, our method would be much simpler and the verification rate is much higher [10, 16, 18]. Since the second round screening could be considered analogous to carrying out a thousand of ELISAs at one time. It would save time and cost.

According to other research findings using LppZ recombinant protein or protein fragments, the positive rate of anti-LppZ antibodies in TB patients was around 30% to 55% [11]. The specificity was not provided. In our result, one peptide was not only sufficient to obtain sensitivity similar to protein component described above, but also with a high specificity (above 90%). Combinative use of two peptides could further improve specificity, but slightly drop of sensitivity. In clinical practice, not every TB suspect is able to take sputum smear test. In our research group, only 56 of 122 TB patients could run sputum smear test and 48 turned positive. In that case, the omission diagnostic rate was actually as high as 60.7% (74/122). In combination of smear test and peptide serology test, the detective rate could improve to 73.0% (Table 5).

In this study, we discover two derived peptides from one protein, which has a diagnostic potential in TB serology test. However, these peptides are not good enough to take place microscopy examine. The present studies provide an advanced strategy of screening TB diagnostic peptide. Integrating multiple diagnostic peptides would improve the existing performance of TB serologic test. Plenty of work had been done on using two or more markers in the form of recombination antigen or antibody groups or others to improve diagnostic efficacy [8, 16, 20, 21]. A research using ESAT-6, CFP10 and PPE68 fusion protein in diagnosing TB could increase the sensitivity to 73.3% [22]. Chemical synthesis of peptides would be a better strategy to study TB diagnostic panel. Peptide array has already revealed application prospect in other disease area. Printed peptide microarray which covering known tumor-associated antigens could predict prognostic in glioblastoma [23]. The array consists of random sequence peptides was capable to describe serum immunosignature pattern and might have potential as a diagnostic tool in Alzheimer’s disease [24]. High-density peptide array may have advanced prospect for comprehensive health monitoring [25]. In our study, we compared the peptide array as a diagnostic tool with the ELISA method. When the cutoff value was set at 4.48 and 3.0 for each pep-LppZ marker, the sensitivity and specificity of peptide array was parallel to ELISA. When we strict the cutoff of peptide arrays to 1.29 and 0.38 for each pep-LppZ marker, the sensitivity of peptide array had a remarkable improve to 75% with not much sacrifice of specificity (Additional file 1: Table S4).

## Conclusions

Our study provides the proof that selected peptides can replace the target proteins in immunodiagnostic test for TB. Peptide array can be a superior tool for screening autoantibody based TB biomarker. High-throughput array technique could perform epitope mapping on a large proteome in an economic and effective way. In this study, two rounds of screening performed well and resulted in satisfactory verification rate. We recommend applying a small amount of sample in the second round of screening. It’s the key point of improving verification rate.

## Methods

### Patients and samples

Serum samples of 187 TB patients and 80 healthy volunteers were obtained from Beijing Chest Hospital Sample Bank (Beijing, China). The serum samples were consecutively collected between January 2012 to December 2014. The clinic characteristics of specimens were sorted in Additional file 1: Table S1. TB patients were diagnosed for pulmonary tuberculosis by either bacteria culture positive or smear positive. None of them were co-infected with HIV. Healthy controls did not have any radiological or clinical signs of TB and had negative tuberculin skin test (TST) results (< 5 mm) and negative IGRA results. Study protocol was approved by the ethics committee of Beijing Chest Hospital, Capital Medical University. The serum sample was prepared according to the standard protocol and stored at − 80 °C until used. All methods were performed in accordance with the relevant guidelines and regulations. The serum pools were consisted with equal volumes of serum from randomly selected 10 TB individuals.

### SPOT synthesis and membranes immunoblotting

Peptide arrays were prepared on amino-PEG500 cellulose membrane-UC540 (Intavis, Germany) using a SPOT robot (Intavis AG, Cologne, Germany) according to standard spot-synthesis protocol. Design program provided with the instrument was used for library designing.

SPOT membranes were equilibrated with PBS-T (phosphate-buffered saline, 0.1% Tween 20, pH 7.4) and blocked with 5% skim milk in PBS-T for 1 h at room temperature. Serum sample was prepared at a dilution of 1:100 in PBS-T with 5% skim milk before incubation of the membranes at 4 °C overnight. After washing with PBS-T, membranes were incubated for 1 h with HRP conjugated anti-Human-IgG (Invitrogen, USA) applied at a dilution of 1:2000 in PBS-T with 5% skim milk.

Measurements of the spot signal intensities were obtained as described [26]. Briefly, the chemiluminescent signals were measured and a digital image file generated. Signal intensities were quantified with TotalLab Software (Nonlinear Dynamics, USA) using algorithms that compared the intensity between background, spot area and negative control to define the empirical probability that the spot signal was distinct from background signal. The positive spot on the membrane was reported as having 100% density, and all other spots had their intensities values expressed as a relative percentage to this intensity. Only spots with intensity values above 30% were considered as positive.

### Overlap peptide library for epitope mapping

To identify the IgG-binding epitopes, a library of 121 peptides was designed to represent and cover the entire sequence (373 amino acids) of LppZ protein (Rv3006). Each peptide was 15 amino acids in length and offset from its neighboring peptide by 3 amino acids. LppZ were assayed against 3 pools of serum samples from TB patients. Each serum pool contained an equivalence mixture of 10 TB serum samples. The peptides were assessed for reactivity with human IgG.

### Dominant epitope screening

16 IgG-bound epitopes of LppZ selected by epitope mapping were integrated on a mini peptide array for dominant epitope screening. Randomly selected 170 TB serum samples and 41 health control samples were used in immunoblotting. In order to normalize between arrays, we spotted FLAG-tag peptide as reference probe on the array membrane. Data of each array was generated by image analysis software including spot gray value and background gray value. The signal value was created after normalization transformation including background correction and baseline conversion.

### Narrow down peptide library for truncation assay

A truncation array of the dominated epitope was performed to determine the minimum length required and key residues for epitope activity. The truncation library was generated through a systematic truncation of the peptide’s sequence from each terminus.

### Quantitative measurement of serum antibodies against dominated epitope by ELISA

For further evaluate the diagnostic effect of these epitopes, ELISA assay were performed. Microtiter 96-well plates were coated with 10 ng/well of purified peptide connected with KLH in a 0.05 M carbonate/bicarbonate buffer (pH 9.6) at 4 °C overnight. The sequence of pep-LppZ-1 and pep-LppZ-13 were S-G-C-A-R-F-N-D-A-Q-S-Q-P-F-T and H-A-H-A-W-A-L-R-M-S-P-D-G-N-V. The plates were blocked with 200 μl of 5% skim milk in PBS-T for 1 h at 37 °C.

Serum sample was prepared at a dilution of 1:100 in PBS-T with 5% skim milk before incubation of the membranes at 4 °C overnight. An equivalence mixed serum pool was served as standards using gradient dilution methods. After washing with PBS-T, the plates were incubated for 1 h with HRP conjugated anti-Human-IgG (Invitrogen, USA) applied at a dilution of 1:2000 in PBS-T with 5% skim milk. Thereafter, the plates were washed with PBS-T and exposed to 100 μl TMB for 20 min at room temperature and stopped by adding 50 μl of 2 M H_2_SO_4_. Absorbance at 450 nm was determined using a spectrophotometer (Epoch Microplate Spectrophotometer, BioTek Laboratories, USA). Each serum sample was tested in duplicate. After normalization by the standards, the levels of serum antibody were calculated to a relative concentration to standards. The cutoff value was choosing when AUC (area under curve) reached max in ROC curve.

### Statistical analysis

Receiver operating characteristic (ROC) curves was utilized to analyze the diagnostic information of each peptide by comparing the area under the curve (AUC). Comparisons of relative concentration of antibodies between groups were preformed using Mann-Whitney U test. Histogram was plotted using GRAPHPAD Prism software (GraphPad Software, Inc., San Diego, CA, USA). Statistically significant differences were those determined to have a *p* value ≤0.05.

## Additional file

Additional file 1: The Supplementary Data. (DOCX 1919 kb)

## Abbreviations

AFB: Acid Fast Bacilli stain
AUC: Area under the curve
IGRA: Interferon Gamma Release Assay
LppZ: Probable conserved lipoprotein LppZ
Mtb: Mycobacterium tuberculosis
ROC: Receiver operator characteristic curve
TB: Tuberculosis
WHO: World Health Organization
