# Supplementary material for: Utility of dominant epitopes derived from cell-wall protein LppZ for immunodiagnostic of pulmonary tuberculosis
Source: BMC Immunol. 2018 Mar 1;19:10. doi: 10.1186/s12865-018-0243-2 (PMC5831716; doi:10.1186/s12865-018-0243-2)

**Utility of dominate epitopes derived from cell-wall protein LppZ for immunodiagnostic of pulmonary tuberculosis**

**Jinjing Tan^1, +^, Xiaoguang Wu^2, +^, Suting Chen^3^, Meng Gu^1^, Hairong Huang^3,^ * and Wentao Yue^1,^***

1 Beijing Chest Hospital, Capital Medical University / Beijing Tuberculosis and Thoracic Tumor Research Institute, Department of Cellular and Molecular Biology, Beijing, 101149, China.

2 Beijing Chest Hospital, Capital Medical University, Department of Tuberculosis, Beijing, 101149, China.

3 Beijing Chest Hospital, Capital Medical University/Beijing Tuberculosis and Thoracic Tumor Institute, National Clinical Laboratory on Tuberculosis, Beijing Key laboratory on Drug-resistant Tuberculosis Research, Beijing, China 101149.

*corresponding author:

Hairong Huang, Huanghairong@tb123.org

Wentao Yue, [yuewt@ccmu.edu.cn](mailto:yuewt@ccmu.edu.cn)

+ these authors contributed equally to this work

Supplementary Table1. Clinical characteristics of specimens.

| **Characteristic** | **TB patients for peptide array**  **(n = 170)** | **Health Controls for peptide array**  **(n= 41)** | **TB patients for ELISA**  **(n = 122)** | **Health Controls for ELISA**  **(n= 78)** |
| --- | --- | --- | --- | --- |
| **Age (years)^1^** | 46.7 ± 19.9 | 24.6 ± 6.2 | 47.1 ± 19.7 | 26.3 ± 6.6 |
| **Male gender** | 115/170 (67.6%) | 12/41 (29.3%) | 81/122 (66.4%) | 23/78 (29.5%) |
| **BCG vaccination^2^** | 129/140 (92.1%) | N.A | 93/101 (92.1%) | N.A |
| **Laboratory findings** |  |  |  |  |
| **Smear positive^3^** | 88/102 (86.3%) | N.A | 48/56 (85.7%) | N.A |
| **Culture positive^4^** | 124/137 (90.5%) | N.A | 88/99 (88.9%) | N.A |
| **IGRA positive^5^** | 114/134 (85.1%) | 41/41 (100%) | 80/93 (86.0%) | 8/78 (10.3%) |

The characteristics showed no statistical differences between peptide array groups and ELISA groups.

1, Age (years): mean ± SD; 2, BCG: Bacillus Calmette- Guerin vaccine; 3, Smear: sputum smear detection for TB bacteria; 4, Culture: bacteria culture; 5, IGRA: IFN-γ release assay (T-SPOT.TB); N.A: not available.

Supplementary Table2. Amino acid sequence of 16 IgG-bound epitopes.

| **Spot name** | **sequence** |
| --- | --- |
| **LppZ-1** | S G C A R F N D A Q S Q P F T |
| **LppZ-2** | A R F N D A Q S Q P F T T E P |
| **LppZ-3** | Q S Q P F T T E P E L R P Q P |
| **LppZ-4** | S S T P P P P P P L P P V P F |
| **LppZ-5** | G A V E E I S I S A E P K V K |
| **LppZ-6** | P V D P A G D G G L M D I V L |
| **LppZ-7** | Y I S T P T D N R V V R V A D |
| **LppZ-8** | P T T A L S G I G S G G G L C |
| **LppZ-9** | T V A V R L A P S T G A V T G |
| **LppZ-10** | V R L A P S T G A V T G E P D |
| **LppZ-11** | E P D V V R K D T H A H A W A |
| **LppZ-12** | K D T H A H A W A L R M S P D |
| **LppZ-13** | H A H A W A L R M S P D G N V |
| **LppZ-14** | A W A L R M S P D G N V W G A |
| **LppZ-15** | L F P Q G G G F P R N N D D K |
| **LppZ-16** | F P Q G G G F P R N N D D K T |

Supplementary Table 3. Statistic characteristics of peptide array signals.

| **Spot name** | **TB patients (n = 170)** | | **Health Controls (n= 41)** | | **P value^1^** | **AUC^2^** |
| --- | --- | --- | --- | --- | --- | --- |
|  | **Mean ± SD** | **Positive rate (cutoff > 30)** | **Mean ± SD** | **Positive rate (cutoff > 30)** |  |  |
| **LppZ-1** | 26.58 ± 81.54 | 35/170 (20.6%) | 1.78 ± 4.38 | 0/41 (0.0%) | <0.001 | 0.795 |
| **LppZ-2** | 24.42 ± 107.20 | 27/170 (15.9%) | 5.93 ± 9.62 | 2/41 (4.9%) | 0.002 | 0.656 |
| **LppZ-3** | 7.57 ± 18.52 | 12/170 (7.1%) | 1.39 ± 2.43 | 0/41 (0.0%) | 0.079 | 0.586 |
| **LppZ-4** | 25.67 ± 122.6 | 20/170 (11.8%) | 1.35 ± 2.46 | 0/41 (0.0%) | <0.001 | 0.698 |
| **LppZ-5** | 7.09 ± 17.18 | 9/170 (5.3%) | 7.58 ± 23.53 | 3/41 (7.3%) | 0.863 | 0.492 |
| **LppZ-6** | 15.30 ± 65.21 | 18/170 (10.6%) | 4.93 ± 9.04 | 1/41 (2.4%) | 0.283 | 0.447 |
| **LppZ-7** | 10.21 ± 23.08 | 17/170 (10.0%) | 0.16 ± 0.95 | 0/41 (0.0%) | <0.001 | 0.830 |
| **LppZ-8** | 9.40 ± 51.74 | 15/170 (8.8%) | 0.51 ± 1.60 | 0/41 (0.0%) | 0.322 | 0.543 |
| **LppZ-9** | 7.09 ± 20.59 | 11/170 (6.5%) | 10.84 ± 15.28 | 7/41 (17.1%) | 0.001 | 0.338 |
| **LppZ-10** | 6.28 ± 16.62 | 11/170 (6.5%) | 1.25 ± 3.14 | 0/41 (0.0%) | 0.035 | 0.598 |
| **LppZ-11** | 5.99 ± 37.63 | 10/170 (5.9%) | 17.08 ± 19.64 | 9/41 (22.0%) | <0.001 | 0.281 |
| **LppZ-12** | 8.38 ± 18.66 | 14/170 (8.2%) | 22.03 ± 111.00 | 3/41 (7.3%) | 0.031 | 0.607 |
| **LppZ-13** | 14.91 ± 27.52 | 26/170 (15.3%) | 0.42 ±1.54 | 0/41 (0.0%) | <0.001 | 0.878 |
| **LppZ-14** | 3.96 ± 11.46 | 8/170 (4.7%) | 0.00 ± 0.00 | 0/41 (0.0%) | <0.001 | 0.712 |
| **LppZ-15** | 16.39 ± 36.82 | 26/170 (15.3%) | 2.39 ± 6.42 | 0/41 (0.0%) | <0.001 | 0.814 |
| **LppZ-16** | 13.80 ± 31.78 | 19/170 (11.2%) | 2.70 ± 6.81 | 1/41 (2.4%) | <0.001 | 0.765 |

1, P value: comparison of difference between TB group and health control group using Mann-Whiney U test; 2, AUC: area under the curve according to receiver operating characteristic (ROC) indicating the discriminatory ability for TB detection from test.

Supplementary Table 4. Comparison of using ELISA and peptide array in TB diagnose.

|  | **pep-LppZ-1** | | | **pep-LppZ-13** | | |
| --- | --- | --- | --- | --- | --- | --- |
| **Subgroups** | **Peptide array**  **(Cutoff > 4.48)** | **Peptide array**  **(Cutoff > 1.29)** | **ELISA**  **(Cutoff > 13.5)** | **Peptide array**  **(Cutoff > 3.0)** | **Peptide array**  **(Cutoff > 0.38)** | **ELISA**  **(Cutoff > 16.3)** |
| **Overall** |  |  |  |  |  |  |
| **Sensitivity** | 54.7% | 75.9% | 49.2% | 57.4% | 82.4% | 43.4% |
| **Specificity** | 90.2% | 80.5% | 83.3% | 90.2% | 92.7% | 88.5% |
| **IGRA negative^1^** |  |  |  |  |  |  |
| **Sensitivity** | 60.0% | 80.0% | 61.5% | 55.0% | 95.0% | 46.2% |
| **Specificity** | 90.2% | 80.5% | 84.3% | 92.7% | 90.2% | 90.0% |

1, IGRA: IFN-γ release assay (T-SPOT.TB).

Supplementary Figure 1. Epitope mapping along the entire sequence of LppZ protein detected in the peptide array with the serum pool.


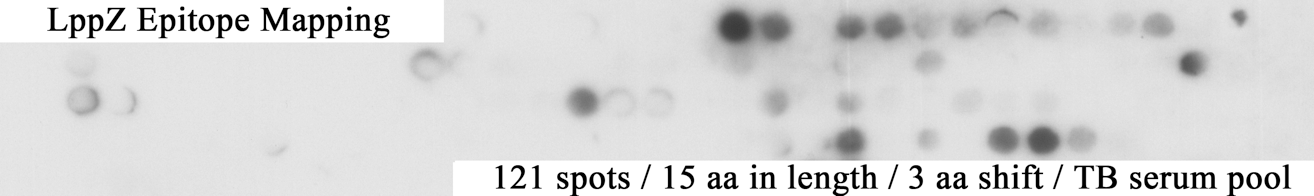


Black box indicated spots synthesized from LppZ protein. IgG-reactive peptides were shown in dark dots.

Supplementary figure 2. Full-length blots image of Figure 1A.


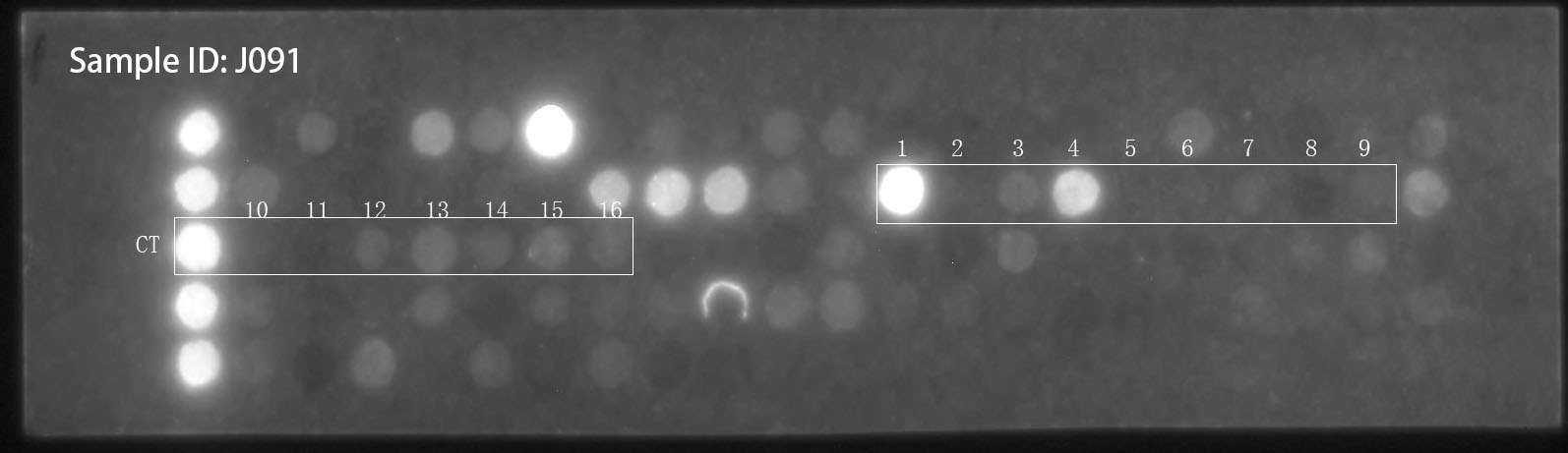


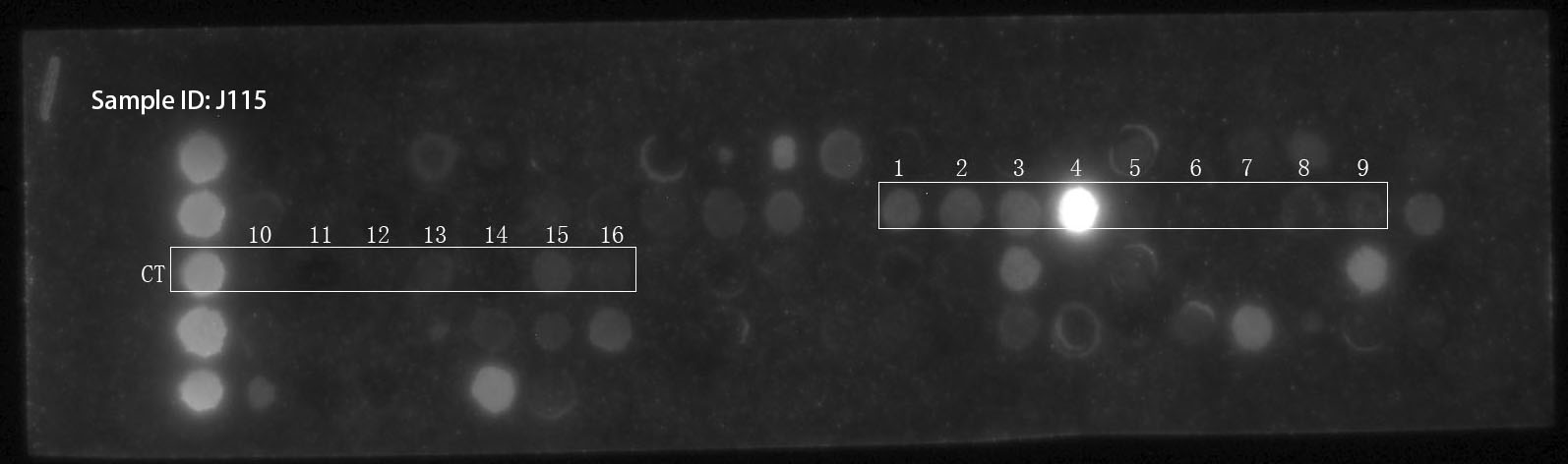


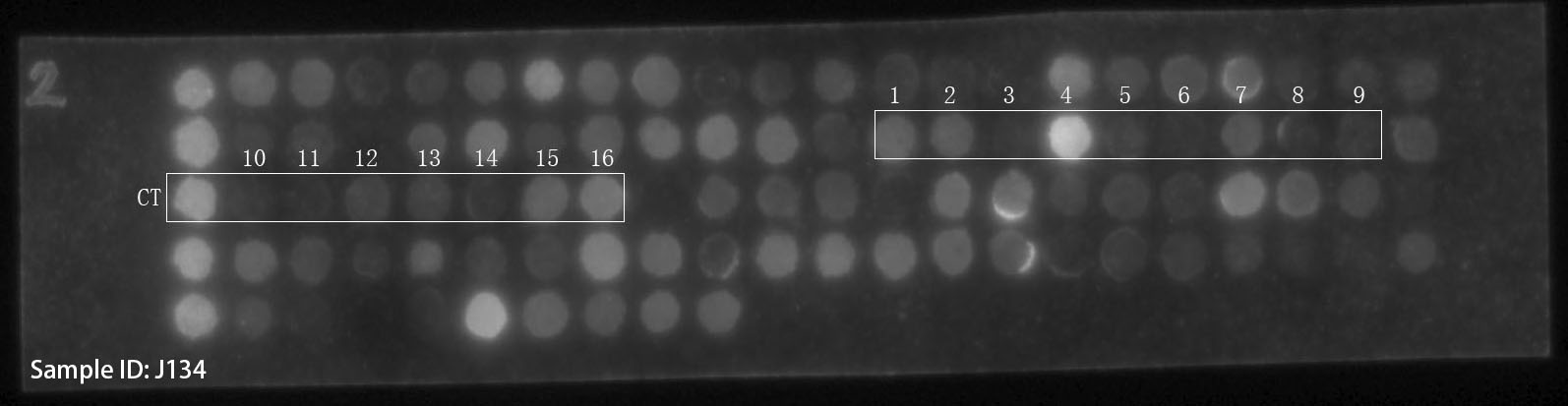


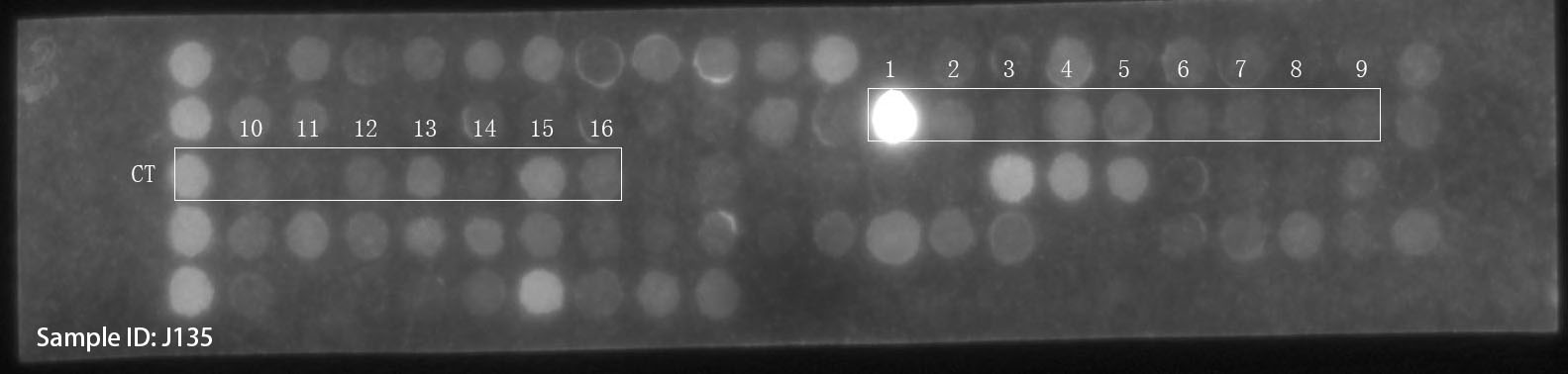


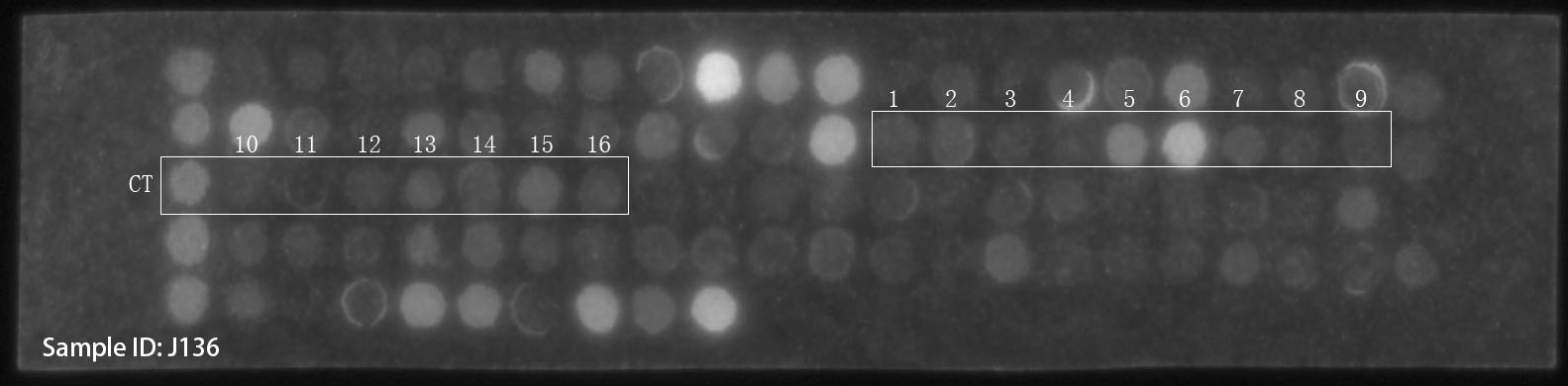


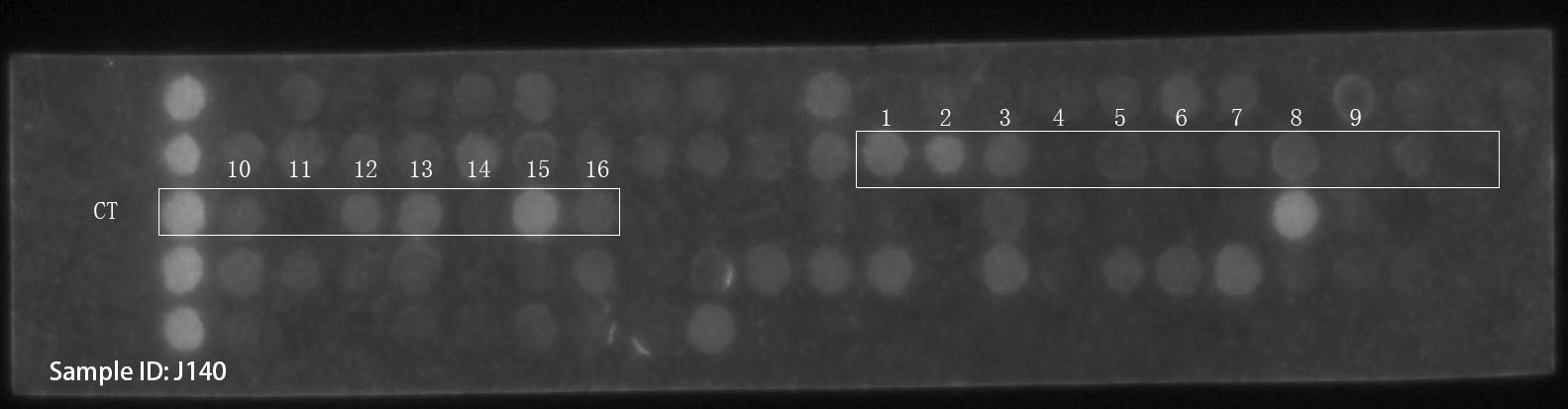


Six immunoblotting image of second round peptide array screening were presented. 16 LppZ epitopes was showed in the box. CT stands for FLAG –tag spot as control spot.

supplementary figure 3. Full-length blots image of Figure 2.


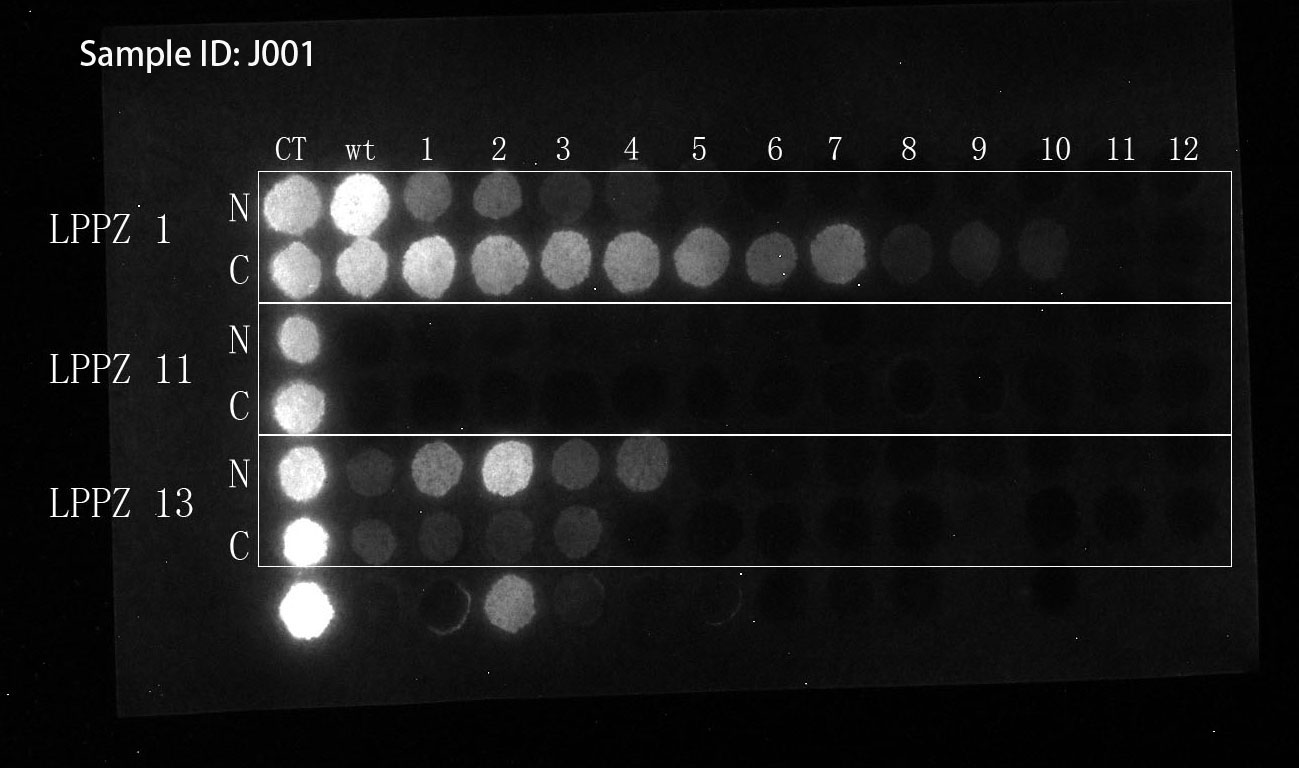


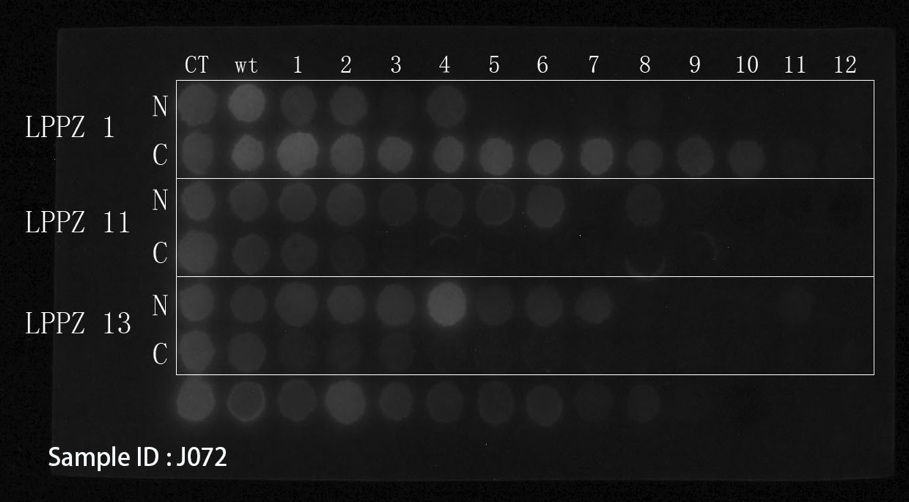


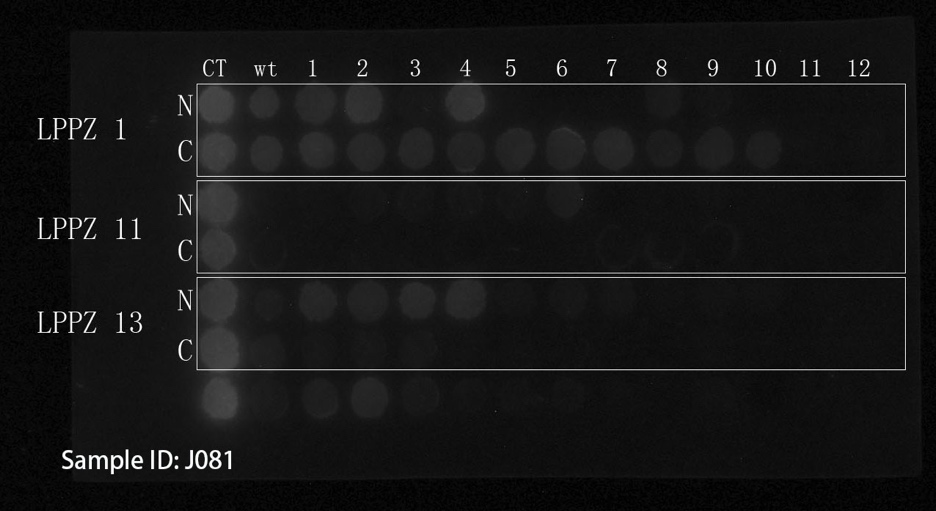


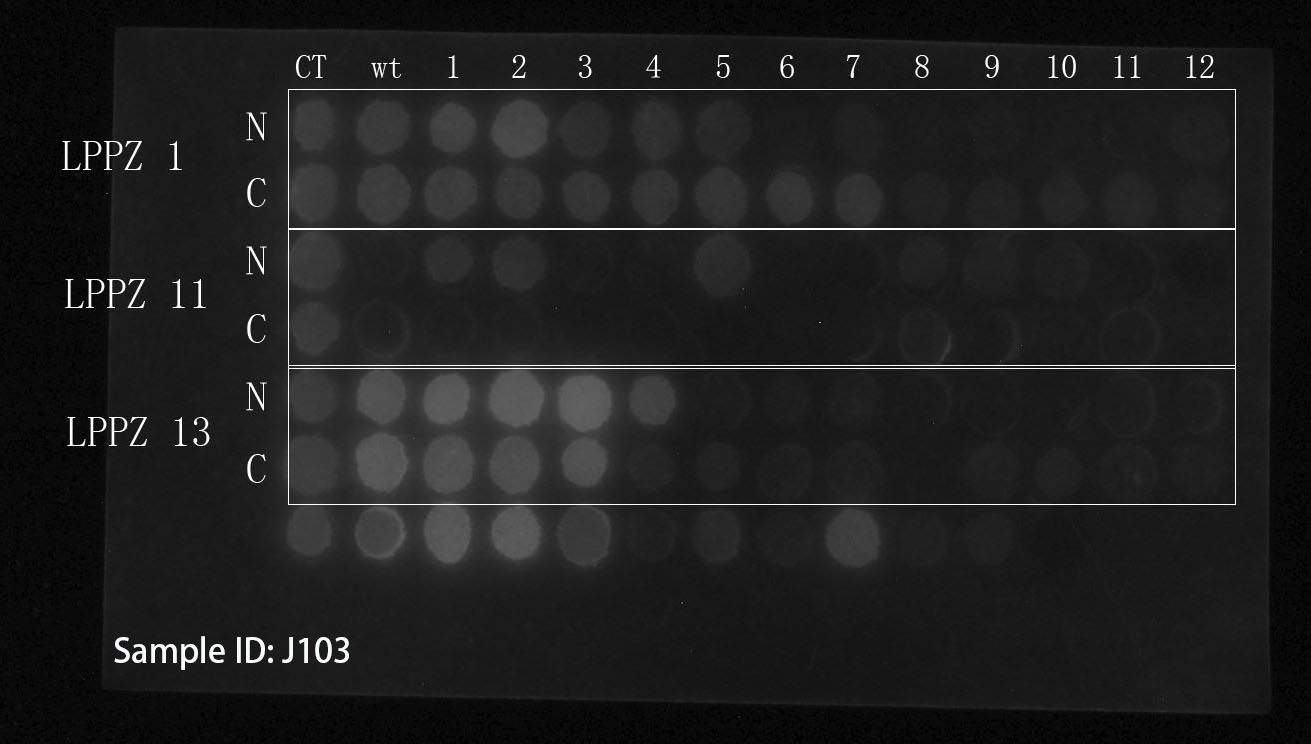


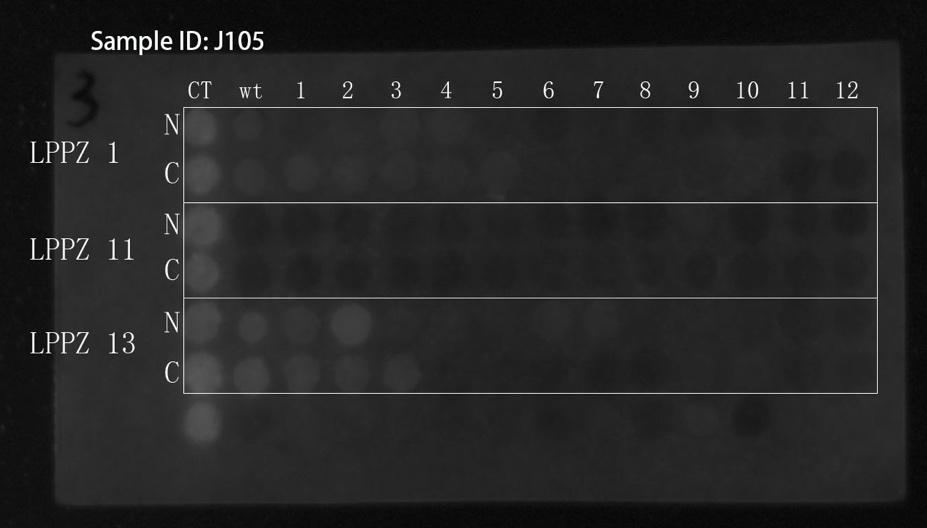

Supplement: Supplementary file 1 — The Supplementary Data. (DOCX 1919 kb) [file 12865_2018_243_MOESM1_ESM.docx]
